# Application of multi-omics technology for the elucidation of anti-pneumococcal activity of 3-acyl-2-phenylamino-1,4-dihydroquinolin-4-one (APDQ) derivative against *Streptococcus pneumoniae*

**DOI:** 10.1038/s41598-020-77694-8

**Published:** 2020-11-26

**Authors:** Sang-Yeop Lee, Hayoung Lee, Sung Ho Yun, Sangmi Jun, Yujeong Lee, Wooyoung Kim, Edmond Changkyun Park, Joonyoung Baek, Yoonna Kwak, Soojin Noh, Giwan Seo, Soojin Jang, Chul Min Park, Seung Il Kim

**Affiliations:** 1 Research Center for Bioconvergence Analysis, Korea Basic Science Institute, Ochang, 28119 South Korea; 2 Convergent Research Center for Emerging Virus Infection, Korea Research Institute of Chemical Technology, Daejeon, 34114 South Korea; 3 Bio-Analytical Science, University of Science and Technology, Daejeon, 34113 South Korea; 4 Center for Research Equipment, Korea Basic Science Institute, Ochang, 28119 South Korea; 5 Department of Toxicology, College of Pharmacy, Chungnam National University, Daejeon, 34134 South Korea; 6 Graduate School of Analytical Science and Technology (GRAST), Chungnam National University, Daejeon, 34134 South Korea; 7 Antibacterial Resistance Research Laboratory, Discovery Biology Department, Institut Pasteur Korea, Seongnam-si, 13488 South Korea

**Keywords:** Antimicrobials, Clinical microbiology

## Abstract

*Streptococcus pneumoniae* is one of Gram-positive pathogen that causes invasive pneumococcal disease. Nowadays, many *S. pneumoniae* strains are resistant to commonly used antibiotics such as β-lactams and macrolides. 3-Acyl-2-phenylamino-1,4-dihydroquinolin-4-one (APDQ) derivatives are known as novel chemicals having anti-pneumococcal activity against *S. pneumoniae*. The underlying mechanism of the anti-pneumococcal activity of this inhibitor remains unknown. Therefore, we tried to find the anti-pneumococcal mechanism of APDQ230122, one of the APDQ derivatives active against *S. pneumoniae*. We performed transcriptomic analysis (RNA-Seq) and proteomic analysis (LC–MS/MS analysis) to get differentially expressed genes (DEG) and differentially expressed proteins (DEP) of *S. pneumoniae* 521 treated with sub-inhibitory concentrations of APDQ230122 and elucidated the comprehensive expression changes of genes and proteins using multi-omics analysis. As a result, genes or proteins of peptidoglycan biosynthesis and DNA replication were significantly down-regulated. Electron microscopy analysis revealed that the structure of peptidoglycan was damaged by APDQ230122 in a chemical concentration-dependent manner. Therefore, we suggest peptidoglycan biosynthesis is a major target of APDQ230122. Multi-omics analysis can provide us useful information to elucidate anti-pneumococcal activity of APDQ230122.

## Introduction

*Streptococcus pneumoniae* is one of Gram-positive human bacterial pathogen that causes invasive pneumococcal disease. Pneumococcal disease encompasses many types of illnesses, including infections of the lungs (pneumonia), the covering around the brain and spinal cord (ear infections, sinus infections, and meningitis), and the bloodstream (bacteremia)^1^. Individuals at high risk for pneumococcal infections include adults aged 65 years or older and children aged 2 years or younger^2^. The World Health Organization (WHO) estimates that each year, 14.5 million episodes of serious pneumococcal disease occur in children under the age of 5, causing up to 500,000 deaths^3^. Although pneumococcal conjugates and polysaccharide vaccines have been used to prevent this disease for more than three decades, the currently marketed vaccines do not cover all serotypes, and prevalent serotype replacement and possible species replacement threatens the long-term use of vaccines^4^.

Antibiotics can be used to treat pneumococcal infections. However, *S. pneumoniae* has continuously developed resistance to commonly used antibiotics such as β-lactams and macrolides. The rate of penicillin and macrolide resistance was more than 30% in the United States, Europe and Asian countries ^5,6^. Increased antibiotic resistance is associated with development of multidrug resistance (MDR) in *S. pneumoniae*. In the United States, 64% of penicillin-nonsusceptible isolates causing invasive pneumococcal diseases were 19A strain, ATCC 700904™, which is highly resistant to antibiotics and exhibits MDR ^7^. According to recent data from the United States, 8% of laboratory-confirmed invasive pneumococcal disease (IPD) is serotype 19A. Among children < 5 years of age, the proportion of IPD caused by serotype 19A is 33%^8^. Nasopharyngeal carriage of non-vaccine *S. pneumoniae* serogroup 15B is increasing, and this serotype may soon become an important cause of pneumococcal disease. Both 15B and 19A were demonstrated to be virulent^9^. Because conjugate vaccines confer imperfect protection against 19F, new antibiotics against *S. pneumoniae* 19F isolates, which are resistant to multiple β-lactam antimicrobials and erythromycin, are urgently needed^10^.

Using a resazurin-based phenotypic assay for high-throughput screening (HTS) of small molecules based on direct measurement of bacterial growth inhibition, Kim et al*.* tried to discover novel antibiotics for treating MDR and extensively drug-resistant *S. pneumoniae* serotypes 15B, 19A, and 19F^11–13^. Primary screening of 27,000 synthetic small molecules from the Korea Chemical Bank (KCB) revealed that several 3-acyl-2-phenylamino-1,4-dihydroquinolin-4-one (APDQ) derivatives had anti-pneumococcal activities against several *S. pneumoniae* strains^13^. Through further optimization studies, we found APDQ 230122 which had excellent antipneumococcal activity with MIC_90_ values of 0.009, 0.019, and 0.033 μM for ATCC 49619 (19F), ATCC BAA1663 (15B), and ATCC 700904 (19A), respectively. APDQ derivatives have been reported to be potent and selective inhibitors of μ-calpain^14^, suppressors of Myc-dependent proliferation of leukemia cells^15^, and potent inhibitors of MERS-CoV activities^16^. Moreover, APDQ compounds inhibit the phosphoserine phosphatases (PSPs) of *Porphyromonas gingivalis* (SerB653), which are crucial for host invasion, intracellular persistence, and innate immune suppression^13,17^.
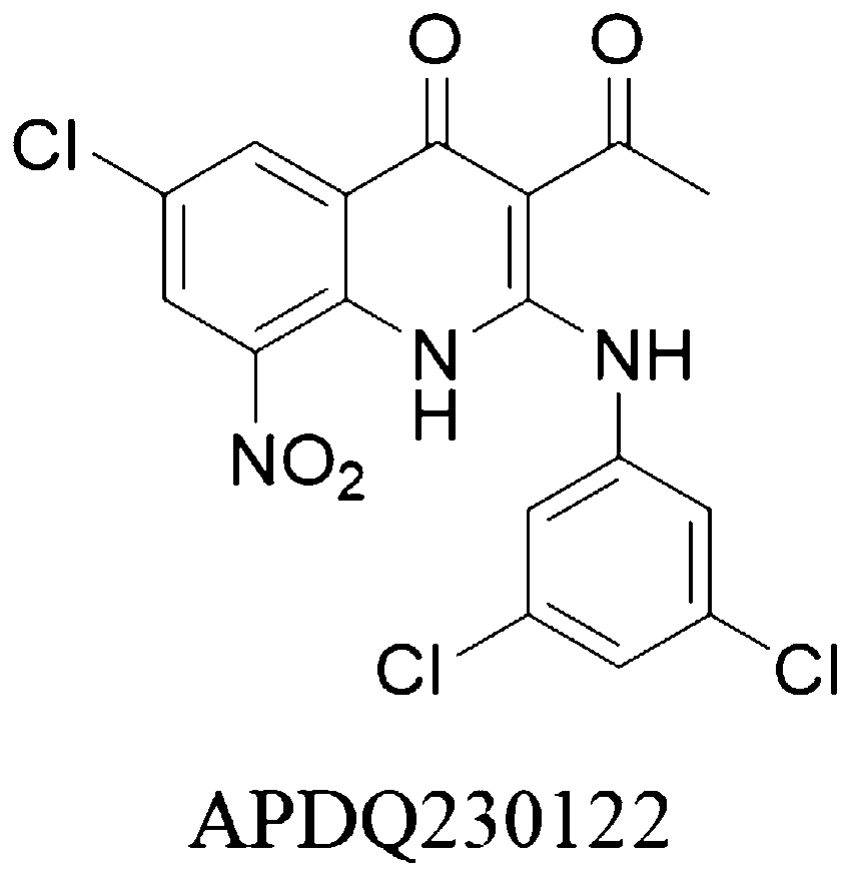


However, the mechanism underlying the anti-pneumococcal activities of this inhibitor remains unknown. In this study, we used multi-omics technologies (genomic, proteomic, and transcriptomic analysis) to elucidate the anti-pneumococcal mechanism of APDQ230122 in *S. pneumoniae*. Our results suggest that APDQ230122 inhibits peptidoglycan biosynthesis in this bacterium.

## Results and discussion

### Antibiotic effect of the APDQ derivative APDQ230122 against *S. pneumoniae* 521

**Figure 1:**
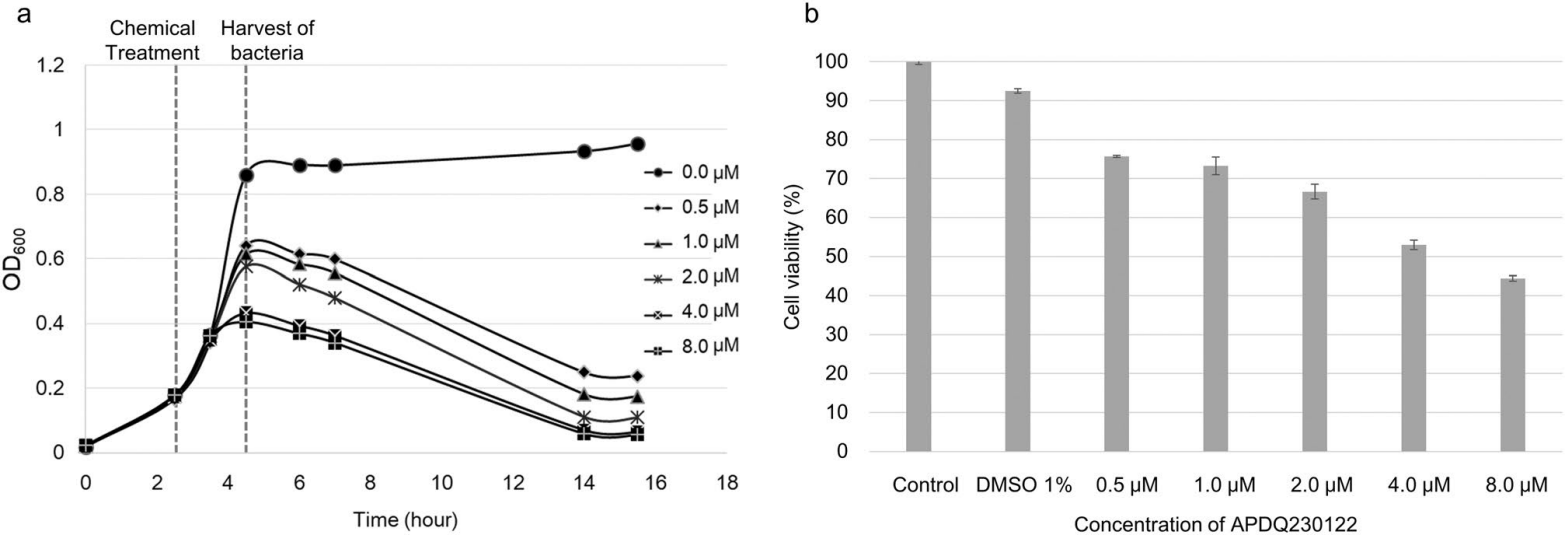
Inhibitory effect of the APDQ derivative APDQ230122 against *S. pneumoniae* 521. (**a**) The clinical strain *S. pneumoniae* 521 was cultured until mid-log phase (≈ OD 0.2) and then treated with the indicated concentrations of APDQ230122 (0.5–8.0 μM). After 2 h, bacteria treated with 1.0 μM APDQ230122 were harvested and used for omics analysis (proteomics analysis and transcriptomics analysis), and non-treated bacteria were used as a control. (**b**) Cell viability tests of *S. pneumoniae* 521 were performed using the same concentrations of APDQ230122 (0.5–8.0 μM). Error bars indicate the SD of the means for four biological replicates.

We treated an antibiotic-sensitive strain with APDQ230122 to reveal the mechanism of antibiotic activity. *S. pneumoniae* 521 (KCTC 43179) is a clinical strain isolated from a Korean hospital; its genome data were deposited on NCBI (accession: CP036529.1). Antibiotic gene screening using ResFinder 3.0 revealed that this strain has no antibiotic resistance genes in its genome. Growth of *S. pneumoniae* 521 was inhibited by 0.5–8.0 μM APDQ230122 (Fig. 1a and Supplementary Table S1). Cell viability decreased in a dose-dependent manner (Fig. 1b) and the concentrations of MIC_50_ and MIC_90_ were determined as 0.5 μM and 2.5 μM, respectively (Supplementary Table S2). Based on these observations, we concluded that APDQ230122 presents bactericidal activity. Because 1.0 μM APDQ230122 was identified as sub-inhibitory concentration in *S. pneumoniae* 521, we prepared *S. pneumoniae* 521 treated with 1.0 μM of APDQ230122 omics analyses.

### Transcriptome (mRNA sequencing) and proteome analysis of *S. pneumoniae* 521

To elucidate the anti-pneumococcal effects of APDQ230122, we performed transcriptomic and proteomic analysis of *S. pneumoniae* 521. In the transcriptomic analysis, we obtained approximately 25.6 million reads on each sample. Among them, an average of 88.9% of filtered reads mapped uniquely to the *S. pneumoniae* 521 genome (Supplementary Table S3). The mRNA levels of 1882 genes are listed in Supplementary Table S4A. KEGG pathway analysis revealed that glycolysis/gluconeogenesis and terpenoid backbone biosynthesis were the major pathways differentially regulated in APDQ230122-treated *S. pneumoniae* 521 (Supplementary Fig. S1). We also performed proteomics analysis to identify expressed proteins and determine protein expression levels. LC–MS/MS analysis identified 1152 and 1142 proteins in non-treated and treated samples, respectively (Supplementary Table S4A).

We combined the proteomic results with the transcriptomic results to search for genes and proteins involved in the antibiotic effects of APDQ230122. For the DEG analysis, 358 genes with *p* < 0.05 were selected from the 1882 genes identified in the transcriptomic data. Among those 358 genes, 170 were upregulated in APDQ230122-treated cells (fold change ≥ 1.5) and 188 genes were downregulated (fold change ≤ − 1.5) (Supplementary Table S4B). For the DEP analysis, we selected 544 of the 1142 proteins identified in the proteomic data. Among those 544 proteins, 168 were upregulated and 376 were downregulated in APDQ230122-treated cells (Supplementary Table S4C).

### Clusters of Orthologous Genes (COG) analysis of *S. pneumoniae* 521

**Figure 2:**
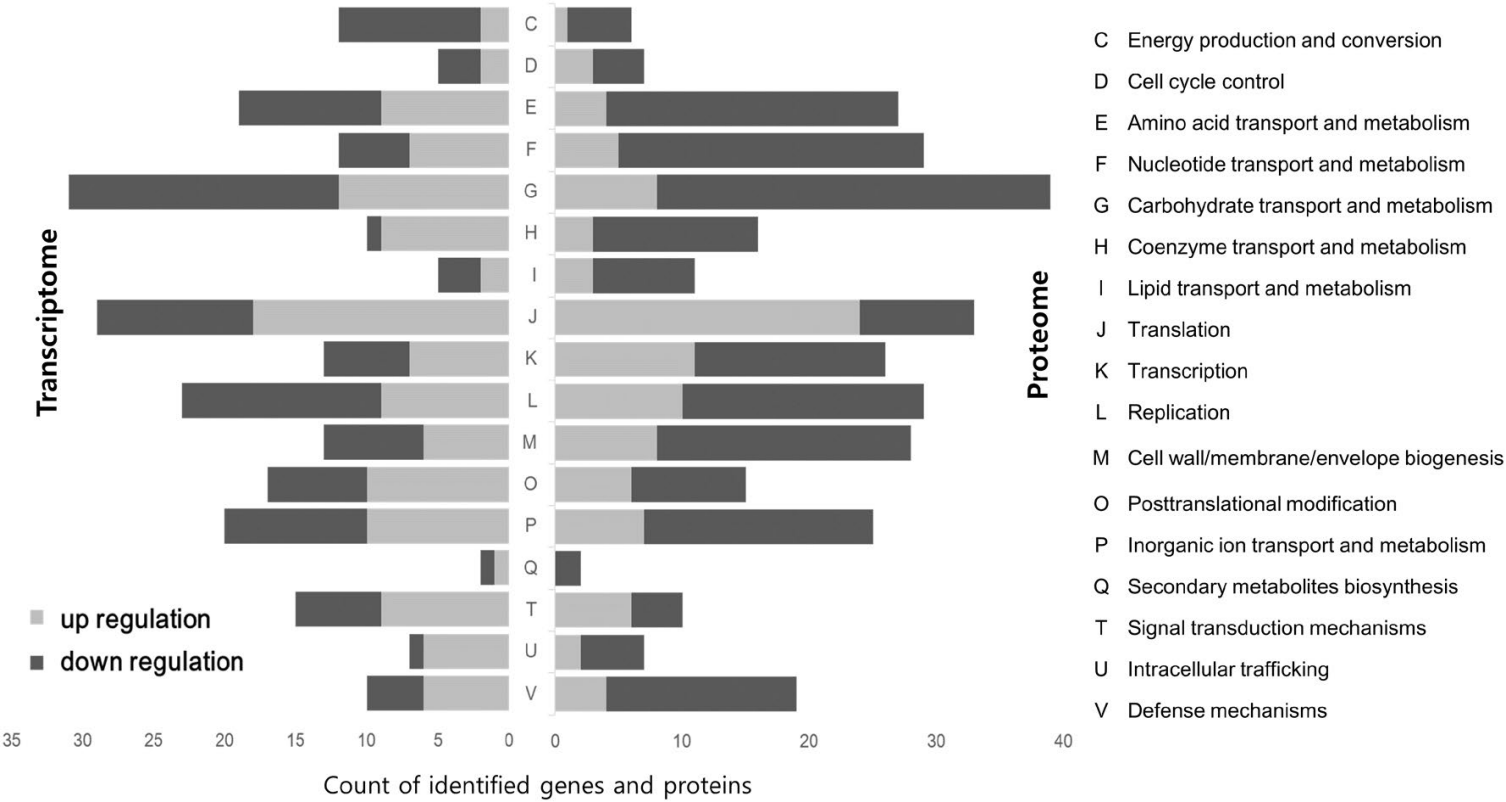
Clusters of Orthologous Groups (COG) analysis of the transcriptome and proteome of drug-treated *S. pneumoniae* 521. Up- and downregulation of COGs are indicated in light gray and dark gray, respectively.

Genes and proteins selected from the DEG and DEP analyses, respectively, were subjected to COG analysis (Fig. 2). In COG analysis using DEG data, we found that many genes involved in energy production and conversion (C), carbohydrate transport and metabolism (G), and replication (L) categories were downregulated in APDQ230122-treated *S. pneumoniae* 521 (Fig. 2). COG analysis using DEP data revealed that downregulation of proteins was more comprehensive: among the downregulated groups were amino acid transport and metabolism (E), nucleotide transport and metabolism (F), cell wall/membrane/envelope biogenesis (M), and defense mechanism (V). Because the biological roles of these categories cover basic metabolic activities and proliferation, our results suggested that treatment with APDQ230122 depresses the basic metabolic activities and proliferation of *S. pneumoniae* 521. One exception was that proteins and genes involved in translation (J) were upregulated under APDQ230122 treatment.

### Application of DEG and DEP analyses to screening of target candidate genes of APDQ230122

The first step toward elucidating the anti-pneumococcal activities of APDQ230122 was to consider well-known antibiotic mechanisms as possible candidate targets. The following eight classes of antibiotic targets were considered: peptidoglycan biosynthesis, fatty acid biosynthetic process, RNA-elongation, DNA-directed DNA polymerase, DNA topoisomerase, DNA gyrase, SOS response, and tRNA ligase^18^. The results of the DEG and DEP analyses were used as a database for screening of candidate target genes of APDQ230122. We identified DEGs and DEPs belonging to eight classes of antibiotic targets, based on Gene Ontology; these are summarized in Table 1. We searched for genes that yielded consistent results in DEGs and DEPs, and found six genes that met this criterion: three involved in peptidoglycan biosynthesis, two involved in fatty acid biosynthesis, and one encoding DNA gyrase (Table 1). In the fatty acid biosynthesis pathway, all but two genes yielded inconsistent results in the transcriptome and proteome data. However, all three genes involved in peptidoglycan biosynthesis were consistently downregulated at the mRNA and protein levels. Therefore, we considered that these proteins involved in peptidoglycan biosynthesis were directly or indirectly inhibited by APDQ230122, and selected peptidoglycan biosynthesis as a potential candidate APDQ230122 target for further analysis.

**Table 1:** Differentially expressed genes and proteins belonging to eight antibiotic targets.

Locus_tag	Protein_id	GeneName	Description	Transcriptome log2 Fold Change (treated/non-treated)	Proteome log ratio (treated/non-treated)
**Peptidoglycan biosynthesis**					
EZ481_RS00200	WP_001227087.1		UDP-*N*-acetylglucosamine 1-carboxyvinyltransferase	**1.268**	0.1219
EZ481_RS02175	WP_000724838.1		UDP-*N*-acetylglucosamine–*N*-acetylmuramyl-(pentapeptide) Pyrophosphoryl-undecaprenol *N*-acetylglucosamine transferase	** − 3.893**	** − 0.2921**
EZ481_RS02180	WP_000863046.1		UDP-*N*-acetylmuramoyl-l-alanine– d-glutamate ligase	** − 3.682**	** − 0.2535**
EZ481_RS06645	WP_000762624.1		Penicillin-binding protein	0.672	**0.3010**
EZ481_RS03695	WP_000470833.1		Phospho-*N*-acetylmuramoyl-pentapeptide-transferase	**1.253**	** − 0.2932**
EZ481_RS08460	WP_000814630.1		d-alanine– d-alanine ligase	− 0.001	** − 0.2967**
EZ481_RS00700	WP_000064394.1	glmU	Bifunctional UDP-*N*-acetylglucosamine diphosphorylase/glucosamine-1-phosphate *N*-acetyltransferase GlmU	0.073	** − 0.2678**
EZ481_RS07490	WP_000370376.1	racE	Glutamate racemase	** − 1.234**	** − 0.2861**
**Fatty acid biosynthetic process**					
EZ481_RS05175	WP_000717456.1	plsX	Phosphate acyltransferase PlsX	** − 1.296**	0.0177
EZ481_RS03340	WP_000167624.1	fabD	ACP S-malonyltransferase	** − 1.513**	− 0.1727
EZ481_RS05170	WP_000136447.1		Acyl carrier protein	** − 3.186**	
EZ481_RS05835	WP_000190397.1		Iron-containing alcohol dehydrogenase	** − 1.301**	
EZ481_RS03320	WP_000565514.1	fabZ	3-Hydroxyacyl-ACP dehydratase FabZ	0.134	0.2106
EZ481_RS03335	WP_000763052.1	fabG	3-Oxoacyl-[acyl-carrier-protein] reductase	0.179	**0.4877**
EZ481_RS03315	WP_000488674.1	accC	Acetyl-CoA carboxylase biotin carboxylase subunit	− 0.616	**0.2679**
EZ481_RS03325	WP_001052244.1		Acetyl-CoA carboxylase biotin carboxyl carrier protein	0.121	**0.4793**
EZ481_RS03355	WP_000852948.1		Ketoacyl-ACP synthase III	0.031	** − 0.2548**
EZ481_RS03935	WP_000649161.1	adhP	Alcohol dehydrogenase AdhP	− 0.357	** − 0.2071**
**RNA elongation**					
EZ481_RS03270	WP_000568640.1	efp	Elongation factor P	**1.850**	0.0535
EZ481_RS02205	WP_000164111.1	typA	Translational GTPase TypA	** − 2.086**	− 0.1712
EZ481_RS09190	WP_000818760.1	greA	Transcription elongation factor GreA	0.409	**0.3722**
**DNA-directed DNA polymerase**					
EZ481_RS01615	WP_000848689.1		Bifunctional DnaQ family exonuclease/ATP-dependent helicase	** − 2.567**	− 0.0458
EZ481_RS06980	WP_001821094.1	rpoC	DNA-directed RNA polymerase subunit beta'	− 0.309	** − 0.5183**
EZ481_RS06975	WP_000907145.1	rpoB	DNA-directed RNA polymerase subunit beta	− 0.087	** − 0.2971**
EZ481_RS00975	WP_000806712.1		DNA polymerase III subunit delta'	0.286	** − 0.3844**
EZ481_RS01195	WP_010976487.1		DNA polymerase III subunit alpha	0.149	** − 0.3522**
**DNA topoisomerase**					
EZ481_RS01400	WP_000037270.1	parE	DNA topoisomerase IV subunit B	**1.267**	0.2596
EZ481_RS01395	WP_001836116.1	parC	DNA topoisomerase IV subunit A	**1.183**	0.1067
**DNA gyrase**					
EZ481_RS01595	WP_000134039.1		DNA gyrase subunit B	** − 3.220**	** − 0.2605**
**SOS response**					
EZ481_RS02525	WP_001061169.1	uvrC	Excinuclease ABC subunit UvrC	**2.958**	0.0442
EZ481_RS05470	WP_000266662.1	recF	DNA replication/repair protein RecF	− 0.362	**0.3680**
EZ481_RS10655	WP_000923570.1	recN	DNA repair protein RecN	0.273	** − 0.3442**
**tRNA-ligase**					
EZ481_RS06140	WP_000546887.1		Tyrosine–tRNA ligase	− 0.847	** − 0.2693**
EZ481_RS01695	WP_001291370.1	metG	Methionine–tRNA ligase	− 0.515	** − 0.2729**
EZ481_RS06285	WP_000031084.1		Glutamate–tRNA ligase	** − 1.591**	− 0.0120
EZ481_RS06055	WP_000830872.1	aspS	Aspartate–tRNA ligase	** − 1.937**	− 0.0670

Genes and proteins belonging to DEG or DEP were bolded.

### Peptidoglycan biosynthesis as a candidate target for APDQ230122 in *S. pneumoniae* 521

**Figure 3:**
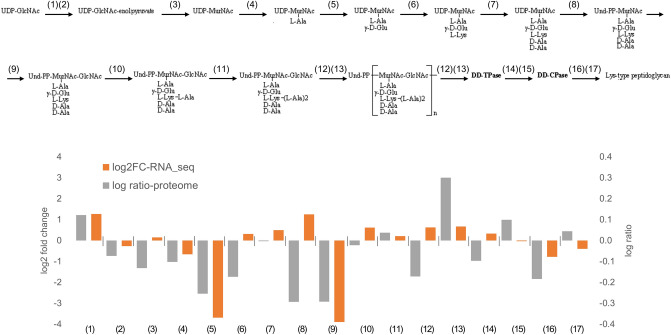
Peptidoglycan synthesis pathway of *S. pneumoniae* 521. Accession numbers of the genes are as follows: (1) EZ481_RS00200, (2) EZ481_RS06950, (3) EZ481_RS09805, (4) EZ481 _RS09170, (5) EZ481_RS02180, (6) EZ481_RS09120, (7) EZ481_RS08465, (8) EZ481_RS03695, (9) EZ481_RS02175, (10) EZ481_RS02540, (11) EZ481_RS02535, (12) EZ481_RS03580, (13) EZ481 _RS06645, (14) EZ481_RS08450, (15) EZ481_RS03700, (16) EZ481_RS01300, (17) EZ48_RS02470. Up- and downregulation of each gene of the peptidoglycan synthesis pathway at the translational and transcriptional levels are indicated by orange and gray bars, respectively.

We identified the genes of the peptidoglycan biosynthesis pathway using blastKOALA on the KEGG website (https://www.kegg.jp/blastkoala/). Seventeen genes involved in the peptidoglycan biosynthesis pathway were identified; their proteomic and transcriptomic data are summarized in Fig. 3 and Supplementary Table S4D. Proteomic results clearly revealed the downregulation of enzymes involved in peptidoglycan biosynthesis. Among the seventeen genes, three [encoding UDP-*N*-acetylmuramoyl-l-alanine-d-glutamate ligase (5), phospho-*N*-acetylmuramoyl-pentapeptide-transferase (8), and UDP-*N*-acetylglucosamine-*N*-acetylmuramyl-(pentapeptide) pyrophosphoryl-undecaprenol *N*-acetylglucosamine transferase (9)] were significantly downregulated. However, two genes [encoding UDP-*N*-acetylmuramoyl-l-alanine-d-glutamate ligase (5) and UDP-N-acetylglucosamine-*N*-acetylmuramyl-(pentapeptide) pyrophosphoryl-undecaprenol *N*-acetylglucosamine transferase (9)] were also remarkably downregulated at the transcriptional level. Future studies should seek to determine whether APDQ230122 functions at the translational or transcriptional level.

### Effects of APDQ230122 on the cell morphology of *S. pneumoniae* 521

**Figure 4:**
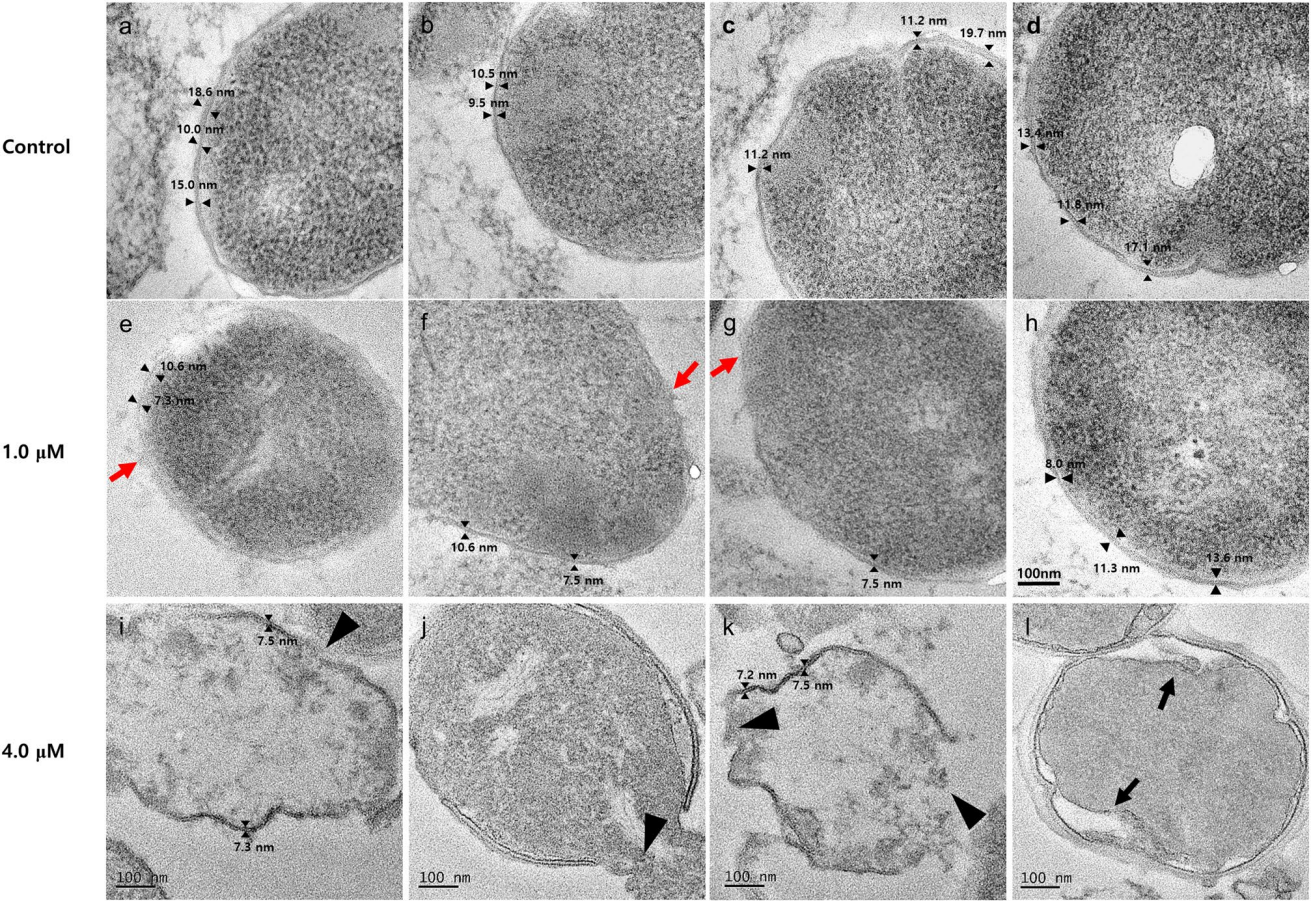
Transmission electron micrographs of *S. pneumoniae* 521 after treatment with the antibacterial chemical APDQ230122. (**a**–**d**) Control cells without treatment had normal shape and peptidoglycan thickness. Cells were treated with (**e**–**h**) 1.0 μM or (**i**–**l**) 4.0 μM APDQ230122. Red arrows, black arrowheads, and arrows indicate damage to *S. pneumoniae* cells in the presence of APDQ230122. Scale bar: 100 nm (for all images).

The strength and cell morphology of gram-positive bacteria such as *S. pneumoniae* are determined by the thickness of the peptidoglycan^19^. To investigate whether APDQ230122 interferes with peptidoglycan biosynthesis in *S. pneumoniae*, we performed TEM to examine the morphology of *S. pneumoniae* 521 cells before and after treatment with APDQ230122. The thickness of cell wall was definitely reduced at 1 μM and 4 μM (*t *test *p *value: 0.008 and 0.006). This results summarized Supplementary Fig. S2. As shown in Fig. 4a–d, the peptidoglycan of non-treated bacteria had a thickness of 13.5 ± 3.6 nm on average (range 9.5–19.7 nm), indicating that the cell wall morphology was well-maintained. By contrast, in bacteria treated with 1.0 μM APDQ230122 (Fig. 4e–h), the peptidoglycan was 9.6 ± 2.3 nm thick (range 7.3–13.6 nm), ~ 30% thinner than in non-treated bacteria. This indicates that APDQ230122 weakens the bacterial peptidoglycan. Moreover, the cell wall was partially lysed (red arrows in Fig. 4e–h). Bacteria treated with 4.0 μM APDQ230122 were more seriously damaged (Fig. 4i–l); In most of the bacteria, their cell walls collapsed and the cytoplasm flowed out of the cell (black arrowheads in Fig. 4i,j,k)^20^. Figure 4l shows that the membranes remained but appeared to be hyperhydrated in the cytoplasm (black arrows)^21,22^.

## Conclusion

APDQ derivatives have shown inhibitory potency on eukaryotic μ-calpain and a few cysteine proteases as well as bacterial phosphoserine phosphatases ^14,17^. Homologues of these enzymes such as peptidase C1 (CGG66885.1) and SerB2 (CJK92665.1) in *S. pneumoniae* might be inhibited by APDQ230122. However, inhibition of these homologues is unlikely responsible for antipneumococcal activity of APDQ230122 that we observed in this study since the enzymes are either not present in our pneumococcal strain (*S. pneumoniae* 521) or dispensable for the bacterial survival in in vitro culture condition^13,23^. Instead, our multi-omics analysis and EM images revealed that APDQ230122 has inhibitory effects on the biosynthesis of peptidoglycan in *S. pneumoniae*. Until, now, however, we could not clearly pinpoint the molecular target of APDQ230122. The results of this study support several assumptions. First, APDQ230122 may directly inhibit peptidoglycan biosynthesis as β-lactams, β-lactamase inhibitors, glycopeptides, d-cycloserine, etc. do ^24^. However, we could not identify a common moiety shared by APDQ230122 and known cell wall biosynthesis inhibitors, suggesting that APDQ230122 may act via different mechanisms, or on different targets, than β-lactam antibiotics. Alternatively, APDQ230122 could induce the downregulation of peptidoglycan biosynthesis at both the translational and transcriptional levels, opening a possibility that transcriptional regulation is the primary target of APDQ230122. Interestingly, APDQ compounds have been reported to inhibit c-Myc transcriptional factor that regulates genes involved in cell-cycle progression and apoptosis in leukemia cells^15^. Proteomic and transcriptomic results revealed that APDQ230122 can also regulate other biological functions, such as replication (Table 1). Several DNA-directed DNA polymerase subunits and DNA gyrase subunits were downregulated by APDQ230122. These replication-related enzymes seemed to be networked with a response regulator transcriptional factor, COG0745, which was also downregulated by APDQ230122 treatment according to PPI analysis (Supplementary Fig. S3). Elucidating the exact biological function of APDQ230122 will require future study, e.g., a high-throughput target mutagenesis study. In summary, our findings demonstrate that multi-omics analysis can yield valuable and comprehensive information about the anti-pneumococcal effects of APDQ230122.

## Methods

### Culture of *S. pneumoniae* 521 and drug treatment

To measure the effect of anti-pneumococcal activity against this bacterium, growth kinetics and cell viability were measured as previously described^13^. Briefly, *S. pneumoniae* 521 (KCTC 43179) was cultivated anaerobically overnight in Tryptic Soy Broth (TSB) at 37.5 °C in an atmosphere containing 5% CO_2_, and then cultured in fresh broth for 7–8 h until mid-log phase (≈OD_600_ 0.2). The culture media were treated with APDQ230122, a 3-acyl-2-phenylamino-1,4-dihydro quinolin-4-one derivative, at the indicated concentrations (0.5–8.0 μM). After 2 h of treatment, treated and non-treated bacteria were harvested, and RNA and protein extracts were prepared. After 4 h of treatment, cell viability testing was tested using the CellTiter-Blue Cell Viability Assay (Promega, Madison, WI, USA). To monitor growth kinetics, *S. pneumoniae* 521 was cultured as described above, and the OD_600_ was measured at each time point.

### RNA sequencing (RNA-Seq) and bioinformatics analysis

Total RNA was isolated using the RNeasy Plus Mini Kit (Qiagen, Valencia, CA, USA) according to the manufacturer’s instructions with minor modifications. RNA quality was assessed by analysis of rRNA band integrity using the Agilent RNA 6000 Nano kit (Agilent Technologies, Santa Clara, CA, USA). Before cDNA library construction, magnetic beads conjugated to oligo(dT) were used to enrich poly (A) mRNA from 2 µg total RNA. Purified mRNAs were disrupted into short fragments, and double-stranded cDNAs were immediately synthesized. After automatic purification using the BluePippin 2% agarose gel cassette (Sage Science, Beverly, MA, USA), suitable fragments were selected as templates for PCR amplification. The final library sizes and qualities were evaluated electrophoretically using the Agilent High Sensitivity DNA kit (Agilent Technologies); average fragment length was between 350 and 450 bp. Subsequently, the library was sequenced on an Illumina NovaSeq6000 sequencer (Illumina, San Diego, CA, USA). Low-quality reads and adapter sequences were filtered out using Trimmomatic (v.0.32)^25^. Filtered reads were aligned to the *S. pneumoniae* 521 genome (accession: NZ_CP036529.1) using STAR (v.2.3.0)^26^. Counts of mapped sequences on genes were calculated using HTSeq-count (v.0.5.4)^27^, and differentially expressed genes (DEGs) were identified using the R package DESeq2^28^. Functional annotations of DEGs were analyzed using GSEA-Pro v.3 (http://gseapro.molgenrug.nl/).

### Sample preparation, in-gel digestion, and proteomic analysis using LC–MS/MS analysis

The harvesting of total protein and preparing for LC–MS/MS were according previously report^29^. Briefly, total extracted protein was fractionated by 12% SDS-PAGE and the fractionated gels were digested by trypsin. The tryptic peptides were enriched and cleaned of chemical contaminants using MGU30-C18 trapping column (LC Packings). And peptides were eluted from the column and directed onto a 10 cm × 75 μm ID C18 reverse-phase column (PROXEON, Odense, Denmark) at a flow rate of 300 nl/min. Peptides were eluted with a gradient of 0–65% acetonitrile over 80 min. All MS and MS/MS spectra were acquired on an LTQ-Velos ESI ion trap mass spectrometer (Thermo Scientific, Waltham, MA, USA) in data-dependent mode. Each full MS (m/z range 400–2000) scan was followed by three MS/MS scans of the most abundant precursor ions in the mass spectrum. MASCOT (v.2.4) was used for the protein identification program. To compare the protein abundance in each sample, we calculated differentially expressed proteins (DEPs) using the log ratio of mol% value with a cut-off value of 0.2.

### Transmission electron microscopy (TEM)

APDQ230122-treated *S. pneumoniae* 521 were pelleted by centrifugation, resuspended in 2.5% glutaraldehyde solution in phosphate buffer (0.1 M, pH 7.4), and incubated for 2 h at 4 °C. The samples were prepared by a method described previously ^30^. The ultrathin plastic sections (80 nm thick) were observed with Zeiss LEO912AB 120 kV TEM (Carl Zeiss) and FEI Tecnai G2 Spirit Twin 120 kV TEM (FEI Company).

## Supplementary information

Supplementary Figures and Tables.

Supplementary Table S4.
