# Supplementary material for: Application of multi-omics technology for the elucidation of anti-pneumococcal activity of 3-acyl-2-phenylamino-1,4-dihydroquinolin-4-one (APDQ) derivative against Streptococcus pneumoniae
Source: Sci Rep. 2020 Nov 26;10:20685. doi: 10.1038/s41598-020-77694-8 (PMC7691496; doi:10.1038/s41598-020-77694-8)
Supplement: Supplementary file 1 — Supplementary Figures and Tables. [file 41598_2020_77694_MOESM1_ESM.pdf]

Application of Multi-omics Technology for the Elucidation of Anti-pneumococcal Activity of 3-Acyl-2-phenylamino-1,4-dihydroquinolin-4-one (APDQ) derivative against *Streptococcus pneumoniae*

Sang-Yeop Lee [1,2,#], Hayoung Lee [1,3,#], Sung Ho Yun [4], Sangmi Jun [2,4] , Yujeong Lee [2,4], Wooyoung Kim [1,2,5], Edmond Changkyun Park [1,2,3], Joonyoung Baek [1,6], Yoonna Kwak [2], Soojin Noh [2], Giwan Seo[1,2], Soojin Jang[7], Chul Min Park [2,*] and Seung Il Kim [1,2,3,*]

[1] Research Center for Bioconvergence Analysis, Korea Basic Science Institute, Ochang, 28119, South Korea

[2] Convergent Research Center for Emerging Virus Infection, Korea Research Institute of Chemical Technology, Daejeon, 34114, South Korea

[3] Bio-Analytical Science, University of Science & Technology, Daejeon, 34113, South Korea

[4] Center for Research Equipment, Korea Basic Science Institute, Ochang, 28119, South Korea

[5] Department of Toxicology, College of Pharmacy, Chungnam National University, Daejeon, 34134, South Korea

[6] Graduate School of Analytical Science and Technology (GRAST), Chungnam National University, Daejeon, 34134, South Korea

[7]Antibacterial Resistance Research Laboratory, Discovery Biology Department, Institut Pasteur Korea, Seongnam-si, 13488, South Korea.

*Corresponding author; Tel:+82-43-240-5422; Fax: +82-43-240-5416; E-mail: ksi@kbsi.re.kr and Tel:+82-42-860-7137; Fax:+82-860-7160; E-mail: parkcm@krict.re.kr

#SYL& HL; equally contributed

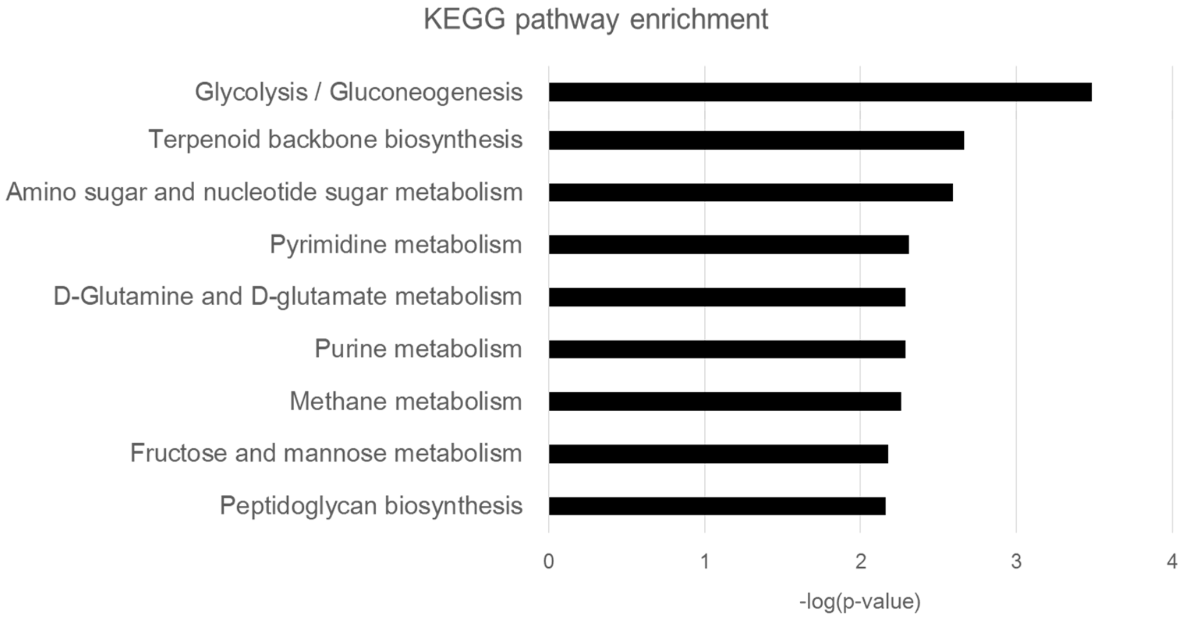

Supplementary Figure S1. Result of KEGG pathway enrichment test in APDQ230122-treated *S. pneumoniae* 521. The top ten enriched metabolic pathways in drug-treated *S. pneumoniae* 521 were selected according to transcriptional level.

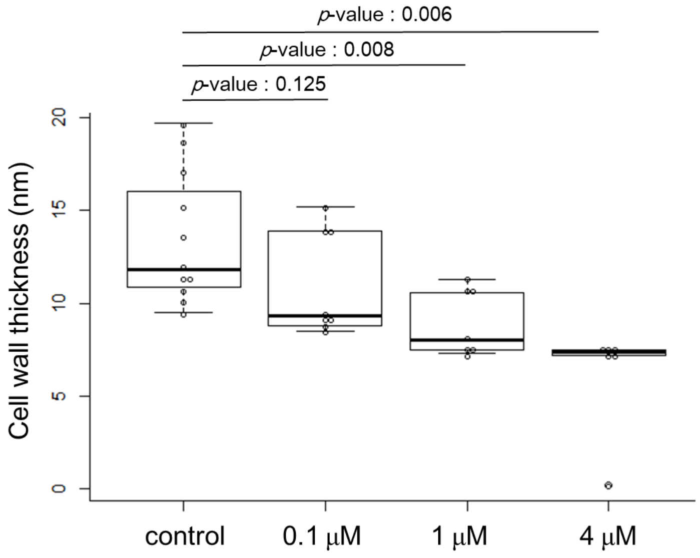

Supplementary Figure S2. Boxplot of cell wall thickness according concentration of APDQ230122. As the concentration of antibiotics increased, the thickness of the cell wall continued to be decreased. When comparing the cell wall thickness between the control and APDQ230122 treated groups, significant difference was detected the concentration of 1 μM.

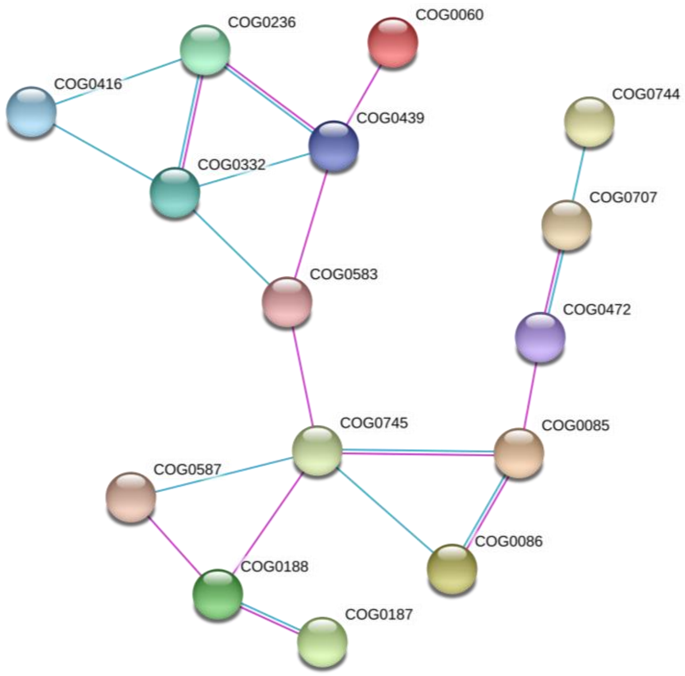

Supplementary Figure S3. Protein-Protein Interaction (PPI) of DEG, DEP, and transcriptional regulator. PPI was constructed by STRING (https://string-db.org/) using active interaction sources of Experiments and Database. COG0745 (response regulator transcription factor) directly interacts with COG0587 (DNA polymerase III subunit alpha), COG0188 (DNA topoisomerase IV subunit A), COG0085 and COG0086 (DNA-directed RNA polymerase subunit beta). The below table shows the information that matches each COG ID.

COG_ID	Locus_tag	Description	log2 Fold Change (treated/non-treated)	log ratio (treated/non-treated)
COG0745	EZ481_RS06225	response regulator transcription factor	-1.15	-0.28
COG0086	EZ481_RS06980	DNA-directed RNA polymerase subunit beta'	-0.31	-0.52
COG0188	EZ481_RS01395	DNA topoisomerase IV subunit A	1.18	0.11
COG0332	EZ481_RS03355	ketoacyl-ACP synthase III	0.03	-0.25
COG0416	EZ481_RS05175	phosphate acyltransferase PlsX	-1.30	0.02
COG0439	EZ481_RS03315	acetyl-CoA carboxylase biotin carboxylase subunit	-0.62	0.27
COG0472	EZ481_RS03695	phospho-N-acetylmuramoyl-pentapeptide-transferase	1.25	-0.29
COG0583	EZ481_RS01015	LysR family transcriptional regulator	-0.62	-0.21
COG0587	EZ481_RS01195	DNA polymerase III subunit alpha	0.15	-0.35
COG0707	EZ481_RS02175	UDP-N-acetylglucosamine--N-acetylmuramyl-(pentapeptide) pyrophosphoryl-undecaprenol N-acetylglucosamine transferase	-3.89	-0.29
COG0744	EZ481_RS06645	penicillin-binding protein	0.67	0.30
COG0085	EZ481_RS06975	DNA-directed RNA polymerase subunit beta	-0.09	-0.30
COG0187	EZ481_RS01400	DNA topoisomerase IV subunit B	1.27	0.26
COG0236	EZ481_RS05170	acyl carrier protein	-3.19	0.00
COG0060	EZ481_RS08520	isoleucine--tRNA ligase	-0.91	-0.16

Supplementary Table S1. Growth rate (OD_{600}) of *S. pneumoniae* 521 according concentration of APDQ230122

Concentration \ hr		0	2.5	3.5 [1]	4.5	6	7	14	15.5
control	1	0.0228	0.1741	0.3598	0.8578	0.8911	0.8895	0.9391	0.9575
	2	0.0165	0.1803	0.3652	0.8555	0.8892	0.8866	0.9213	0.9575
	3	0.0166	0.1854	0.373	0.8693	0.8929	0.8923	0.9407	0.956
0.5 μM	1	0.0185	0.16	0.326	0.6305	0.607	0.588	0.2186	0.2108
	2	0.0207	0.174	0.3593	0.6527	0.6223	0.6094	0.2795	0.2658
	3	0.0144	0.1881	0.3947	0.7365	0.7072	0.6975	0.5675	0.5659
1 μM	1	0.024	0.159	0.3201	0.5809	0.5497	0.5218	0.1387	0.1348
	2	0.0221	0.1749	0.3553	0.606	0.5721	0.5423	0.1478	0.1444
	3	0.0199	0.1959	0.4119	0.6547	0.6283	0.6055	0.2585	0.2437
2 μM	1	0.0165	0.1578	0.3187	0.5519	0.4975	0.4566	0.0969	0.094
	2	0.0226	0.175	0.3546	0.5725	0.5127	0.4665	0.1057	0.1033
	3	0.0251	0.1894	0.3841	0.6045	0.5553	0.5157	0.1311	0.1296
4 μM	1	0.0213	0.1604	0.3195	0.3547	0.3255	0.3066	0.0652	0.0513
	2	0.0201	0.1704	0.345	0.447	0.4066	0.38	0.0662	0.0628
	3	0.0229	0.1873	0.3832	0.4969	0.4482	0.4031	0.0793	0.0774
8 μM	1	0.0225	0.1647	0.3374	0.3867	0.3488	0.3212	0.0528	0.0489
	2	0.0225	0.1775	0.365	0.4168	0.3858	0.3545	0.063	0.0593
	3	0.023	0.1922	0.3875	0.4129	0.3742	0.3464	0.0612	0.0583

[1] APDQ stock solution was added into each culture media (final concentration of 0.5μM - 8μM) after 3.5h incubation.

Supplementary Table S2. MIC_{50} and MIC_{90} of APDQ 230122 in *S. pneumoniae* 521

		MIC (μM)		
Antibiotics	Replicate	MIC_{50}	MIC_{90}	Screening range
APDQ 230122	5	0.5	2.5	0.1~20

Minimum inhibitory concentrations (MICs) of archived clinical respiratory isolates of *S. pneumoniae* 521 to APDQ 230122 were determined by broth dilution method following incubation at 48 hours. The colony forming units (CFUs) cultured on the agar plates were counted to determine MICs [supp1].

Supplementary Table S3. Results of RNA-Seq alignment

	Control 1st	Control 2nd	Control 3rd	1μm of APDQ 1st	1μm of APDQ 2nd	1μm of APDQ 3rd
Reads	26,552,910	27,299,058	26,073,394	30,370,207	23,882,586	17,433,894
Uniquely mapped reads	24,274,136	24,306,162	23,720,966	26,674,790	20,619,171	15,307,705
Uniquely mapped reads %	91.42%	89.04%	90.98%	87.83%	86.34%	87.80%

Supplementary Reference

Supp1. Petra L. *et al*. Comparison of Broth Microdilution, E Test, and Agar Dilution Methods for Antibiotic Susceptibility Testing of Campylobacter jejuni and Campylobacter coli. Journal of Clinical Microbiology 41, 1062-1068.
